# Long-term outcomes and dynamic changes of in-stent stenosis after Pipeline embolization device treatment of intracranial aneurysms

**DOI:** 10.1136/jnis-2022-019680

**Published:** 2023-01-23

**Authors:** Siming Gui, Xiheng Chen, Dachao Wei, Dingwei Deng, Wei You, Xiangyu Meng, Jian Lv, Junqiang Feng, Yudi Tang, Shu Yang, Ting Chen, Peng Liu, Huijian Ge, Hengwei Jin, Xinke Liu, Yuhua Jiang, Wei Feng, Youxiang LI

**Affiliations:** 1 Beijing Neurosurgical Institute, Capital Medical University, Beijing, China; 2 School of Biomedical Engineering, Capital Medical University, Beijing, China; 3 Department of Interventional Neuroradiology, Beijing Tiantan Hospital, Beijing, China; 4 Department of Epidemiology and Health Statistics, Capital Medical University, Beijing, China

**Keywords:** Flow Diverter, Aneurysm, Complication, Intervention, Stenosis

## Abstract

**Background:**

Flow diverters have revolutionized the treatment of intracranial aneurysms. However, the delayed complications associated with flow diverter use are unknown.

**Objective:**

To evaluate the incidence, severity, clinical outcomes, risk factors, and dynamic changes associated with in-stent stenosis (ISS) after treatment with a Pipeline embolization device (PED).

**Methods:**

Patients who underwent PED treatment between 2015 and 2020 were enrolled. The angiographic, clinical, and follow-up data of 459 patients were independently reviewed by four neuroradiologists to identify ISS. Binary logistic regression was conducted to determine ISS risk factors, and an ISS–time curve was established to demonstrate dynamic changes in ISS after PED implantation.

**Results:**

Of the 459 treated patients, 69 (15.0%) developed ISS. At follow-up, nine patients (2.0%) with ISS demonstrated reversal, while 18 (3.9%) developed parental artery occlusion. A total of 380 patients (82.8%) achieved complete aneurysm occlusion (O’Kelly–Marotta grade D). Patients with posterior-circulation aneurysm (OR=2.895, 95% CI (1.732 to 4.838; P<0.001) or balloon angioplasty (OR=1.992, 95% CI 1.162 to 3.414; P=0.037) were more likely to develop ISS. Patients aged >54 years (OR=0.464, 95% CI 0.274 to 0.785; P=0.006) or with a body mass index of >28 kg/m^2^ (OR=0.427, 95% CI 0.184 to 0.991; P=0.026) had a lower ISS risk. Intimal hyperplasia initiated by PED placement peaked within 1 year after the procedure, rarely progressed after 12 months, and tended to reverse within 24 months.

**Conclusions:**

ISS is a common, benign, and self-limiting complication of PED implantation in the Chinese population.

**WHAT IS ALREADY KNOWN ON THIS TOPIC**

- In-stent stenosis (ISS) after Pipeline embolization device (PED) implantation is a common and self-limiting complication.
- ISS is a long-term complication after PED implantation. However, the outcomes, dynamic changes, and risk factors for ISS remain unknown.

**WHAT THIS STUDY ADDS**

- Patients with posterior-circulation aneurysm and balloon angioplasty are more likely to develop ISS. ISS reaches its peak at 6–12 months, and the progression is reversed within 24 months.

**HOW THIS STUDY MIGHT AFFECT RESEARCH, PRACTICE OR POLICY**

- We strongly recommend at least 2 years of postoperative angiographic follow-up to identify and evaluate ISS dynamically in patients undergoing PED placement.

## Introduction

Flow diverter (FD) treatment in patients with intracranial aneurysm is widely used because of its safety and high aneurysm occlusion rate.^1–5^ Although FDs have been available for over a decade, the complications associated with their implantation have not been thoroughly examined, especially in terms of long-term prognosis. Frequently studied complications after FD treatment include perioperative ischemic and hemorrhagic events.^6–11^ However, other long-term complications after FD treatment, such as in-stent stenosis (ISS), have received less attention in terms of their clinical manifestation and pathology. Previous studies have shown that ISS is a rare complication after FD implantation.^12–18^ However, this complication is difficult to analyze statistically because of the very small number of total cases and lack of long-term dynamic follow-up data to evaluate the changes in ISS. Thus, the incidence and clinical and angiographic outcomes, the dynamic changes in ISS after Pipeline embolization device (PED) implantation, and the risk factors for ISS require further evaluation. In the present study, we evaluated the incidence, clinical consequences, risk factors, and dynamic changes in ISS based on postoperative follow-up after PED treatment.

## Methods

### Study design and patients

In this study, all consecutive patients with intracranial aneurysm who underwent embolization using the PED (Covidien, Irvine, California, USA) at our hospital were prospectively maintained in a database between September 1, 2015, and October 31, 2020. All patients provided written informed consent for the use of any identifiable patient photographs. The study protocol was reviewed and approved by the ethics committee, and the study adhered to the principles of the Declaration of Helsinki.

All patients underwent conventional catheter angiography during follow-up, and all angiographic images for ISS assessment were acquired from digital subtraction angiography data. Exclusion criteria included patients (1) who lacked follow-up imaging data or who were uncontactable during follow-up, (2) with missing data on demographics, aneurysm characteristics, antiplatelet regimen, procedure details, or neurological complications in the electronic medical record system, (3) with aneurysms related to arteriovenous malformations, or (4) who were unable to undergo antiplatelet aggregation therapy. A study flow chart of patient selection is shown in online supplemental figure 1.

10.1136/jnis-2022-019680.supp1 Supplementary data

### Definition of stenosis rate

The stenosis rate (SR) was defined using the following equation:

$$SR=\left(1-\frac{No.n\;follow-up\;In-stent\;artery\;Diameter\left(Dn\right)}{operation\;In-stent\;artery\;Diameter\left(D0\right)}\right)\times 100\%$$

### Definition of ISS

ISS was defined as a stenosis rate (SR) of ≥50% at angiographic follow-up, irrespective of whether the SR was <50% (recovery) at subsequent follow-up. According to the SR, the stenosis grade was classified as mild (50%≤SR<70%), severe (70%≤SR<99%), or parental artery occlusion (SR=100%).

### Definition of anterior-circulation aneurysms and posterior-circulation aneurysms

The definitions of anterior-circulation aneurysms and posterior-circulation aneurysms are shown in online supplemental material.

### Definition of aneurysm and parental artery morphology parameters

The morphology parameters assessed in this study were defined based on previous studies^19 20^ and are described in the online supplemental material.

### Procedures

Dual antiplatelet therapy (DAPT) with aspirin (100 mg) combined with clopidogrel (75 mg) was administered at least 5 days prior to the procedure. All patients underwent platelet function testing at 1 day before PED placement. The protocol for platelet function testing is described in the online supplemental material. For non-responders, clopidogrel was switched to ticagrelor (180 mg once) before the procedure. Generation of the PED, the PED implantation procedure, and the duration and type of DAPT after the procedure are shown in the online supplemental material.

### Follow-up and outcomes

Clinical follow-up was conducted at 3 months, 6 months, 1 year, 2 years, and occasionally longer after the PED implantation. All postoperative complications and medication compliance were recorded at follow-up. Aneurysm occlusion and morphology parameters were measured using digital subtraction angiography, and the occlusion grading was established based on the O’Kelly–Marotta grading scale.^21^ The first angiography follow-up was conducted at 3–6 months after the PED implantation. The study outcomes included ISS occurrence, postprocedural ischemic or hemorrhagic events, and aneurysm occlusion. The status of the parental artery was also evaluated. Four neuroradiologists reviewed the imaging and endpoint events. In patients where the evaluation result was disputed, the team reached a unanimous decision after a discussion. The person in charge of the review committee supervised the reading procedures and was responsible for the accuracy and validity of the conclusions.

### Statistical analysis

Continuous variables, such as demographic data, clinical records, aneurysm characteristics, and follow-up imaging results, are described as mean±SD or median (IQR), as appropriate, according to the data distribution (normality was proved using histograms and Q–Q plots). Categorical variables are expressed as frequency (percentage). Comparative analyses between the ISS and non-ISS groups were performed using the t-test and the non-parametric Mann–Whitney U test for continuous variables, the non-parametric Mann–Whitney U test for ordinal variables, and the Χ^2^ test or Fisher’s exact test for categorical variables. The univariate logistic regression analysis was conducted to test the relationship between ISS and the variables mentioned above. Covariates with P values of <0.1 in the univariate analysis were entered into the multivariate analysis using the backward stepwise selection method. All statistical analyses, including analyses to obtain crude and adjusted ORs and 95% confidence intervals, were performed using R (v4.1.1 and above; R Foundation for Statistical Computing, Vienna, Austria). All P values were based on two-tailed statistical tests, and P values <0.05 were considered statistically significant.

## Results

### Patient and follow-up characteristics

Between September 2015 and December 2020, a total of 459 consecutive patients (median age, 54 years (IQR 47–61 years); men, 151 (32.9%)) treated with the PED were included in this study (table 1). Sixty-nine patients (15.0%) developed ISS in the follow-up period. The median follow-up period was 9 months (IQR 6–16 months) (table 2). ISS spontaneously recovered in nine patients on final angiographic follow-up. The other 60 patients experienced ISS, 32 (53.3%) of whom developed mild ISS, 10 (16.7%) of whom developed severe ISS, and 18 (30%) of whom developed parental artery occlusion. All cases of complete parental artery occlusion were detected during the follow-up period. Subgroup analysis (online supplemental material) revealed a significant difference between the ISS resolution group and the ISS non-resolution group (25 (14–36) months vs 14 (9–20) months, respectively; P=0.005) in the mean follow-up duration of patients with ISS. Of the 69 patients with ISS, four (5.8%) experienced hemiplegia, one (1.4%) experienced hemianopia, and two (2.9%) died from brainstem infarction because ISS developed into basilar artery occlusion. The number of patients with a body mass index (BMI) of ≥28 kg/m^2^ in the non-ISS group (n=64 (16.4%)) was significantly higher than that in the ISS group (n=4 (5.8%)) (P=0.022).

**Table 1:** Baseline characteristics of patients after Pipeline embolization device (PED) treatment

Characteristics	Non-ISS (n=390)	ISS (n=69)	Total (n=459)	P value
Stenosis grade (last follow-up) (non-ISS=390, ISS=60)				–
No stenosis (SR<50%)	399 (100%)	0 (0%)	399 (86.9%)	
Mild stenosis (50%≤SR<70%)	0 (0%)	32 (53.3%)	32 (7.0%)	
Severe stenosis (70%≤SR<99%)	0 (0%)	10 (16.7%)	10 (2.2%)	
Occlusion (SR=100%)	0 (0%)	18 (30%)	18 (3.9%)	
Age	54 (48–61)	52 (46–52)	54 (47–61)	0.117
Male	122 (31.3%)	29 (42.0%)	151 (32.9%)	0.080
Height (cm)	164 (160–170)	165 (160–170)	164 (160–170)	0.443
Weight (kg)	65 (59–75)	68 (60–75)	65 (59–75)	0.764
BMI	24.61 (22.53–26.95)	24.46 (23.14–26.42)	24.56 (22.67–26.73)	0.935
Fat (BMI≥28 kg/m^2^)	64 (16.4%)	4 (5.8%)	68 (14.8%)	**0.022**
Diabetes mellitus	35 (9.0%)	6 (8.7%)	41 (8.9%)	0.940
Dyslipidemia	163 (41.8%)	25 (36.2%)	188 (41.0%)	0.386
Hypertension	165 (42.3%)	29 (42.0%)	194 (42.3%)	0.577
Pre-SAH	20 (5.1%)	3 (4.3%)	23 (5.0%)	1.000
Pre-ischemic stroke	28 (7.2%)	4 (5.8%)	32 (7.0%)	0.874
Coronary heart disease	28 (7.2%)	6 (8.7%)	34 (7.4%)	0.658
Current smoking	61 (15.6%)	13 (18.8%)	74 (16.1%)	0.505
Regular alcohol drinkers	55 (14.1%)	11 (15.9%)	66 (14.4%)	0.161
Allergic history	52 (13.3%)	6 (8.7%)	58 (12.6%)	0.285
Preinterventional operation	25 (6.4%)	3 (4.3%)	28 (6.1%)	0.699

BMI, body mass index; ISS, in-stent stenosis; SAH, subarachnoid hemorrhage; SR, stenosis rate.

**Table 2:** Follow-Up characteristics of patients after Pipeline embolization device (PED) treatment

Characteristics	Non-ISS (n=390)	ISS (n=69)	Total (n=459)	P value
Postoperative complications	5 (1.3%)	3 (4.3%)	8 (1.7%)	0.195
First follow-up time	7 (5–9)	6 (5–7)	6 (5–8)	**0.026**
First follow-up parental artery diameter	3.12 (2.60–3.66)	1.65 (0.50–2.32)	2.95 (2.32–3.53)	**<0.001**
First follow-up aneurysm complete occlusion	306 (78.5%)	53 (76.8%)	359 (78.2%)	0.760
Last follow-up time	9 (6–16)	12 (6–19)	9 (6–16)	0.116
Last follow-up parental artery diameter	3.12 (2.55–3.66)	1.16 (0–1.80)	2.90 (2.26–3.56)	**<0.001**
Last follow-up aneurysm complete occlusion	320 (82.1%)	60 (87.0%)	380 (82.8%)	0.320
Severe postoperative symptoms* related to ISS	0 (0%)	5 (7.2%)	5 (1.1%)	–
Mortality related to ISS (basilar artery occlusion)	0 (0%)	2 (2.9%)	2 (0.4%)	–
Drug withdrawal	82 (21.0%)	21 (30.4%)	103 (22.4%)	0.116
Clopidogrel switched to ticagrelor	58 (14.9%)	14 (20.3%)	72 (15.7%)	0.254

*Severe postoperative symptoms included hemidysesthesia, hemiplegia, and hemianopia.

ISS, in-stent stenosis.

There was no difference in the rate of postoperative complications (ischemic or hemorrhagic) between the non-ISS and ISS groups (1.3% vs 4.3%, respectively; P=0.195). A total of 380 patients (82.8%) achieved complete occlusion after stent implantation. There were no differences in the proportion of patients attaining a favorable outcome between the non-ISS group (82.1%) and the ISS group (87%) (P=0.32). There were no differences in the rate of antiplatelet drug withdrawal (21.0% vs 30.4%, respectively; P=0.116) or the change in antiplatelet therapy (1.8% vs 2.9%, respectively; P=0.630) between the non-ISS and the ISS groups.

### Aneurysm characteristics

We included the aneurysm characteristics of 459 patients in our statistical analysis (online supplemental table 1). Aneurysms in the non-ISS group were more frequently located in the anterior circulation, while the proportion of aneurysms in the anterior circulation was lower in the ISS group (81.9% vs 63.8%, respectively; P=0.008). The proportion of patients who underwent balloon angioplasty was significantly different between the non-ISS and the ISS groups (17.4% vs 30.4%, respectively; P=0.012). The proportion of patients treated with the PED with coiling was similar between the non-ISS and ISS groups (37.4% vs 42%, respectively; P=0.469). Certain angiographic parameters, including aneurysm neck (6.69 mm (IQR 4.45–11.10) vs 10.10 mm (IQR 6.52–15.75), respectively; P<0.001), aneurysm maximum diameter (10.40 mm (IQR 6.29–16.70) vs 13.3 mm (IQR 9.07–22.20), respectively; P=0.003), aneurysm height (7.37 mm (IQR 4.76–11.80) vs 7.87 mm (IQR 6.57–14.45), respectively; P=0.025), aneurysm width (8.48 mm (IQR 4.81–14.63) vs 12.90 mm (IQR 6.80–20.95), respectively; P<0.001), aneurysm perpendicular height (6.96 mm (IQR 4.50–11.23) vs 7.87 mm (IQR 5.83–14.25), respectively; P=0.017), height/width ratio (0.89 (IQR 0.74–1.02) vs 0.81 (IQR 0.64–0.95), respectively; P=0.012), size ratio (2.87 (IQR 1.64–4.73) vs 3.56 (IQR 2.18–6.32), respectively; P=0.007), and neck ratio (1.91 (IQR 1.15–3.02) vs 2.93 (IQR 1.56–4.40), respectively; P<0.001), were significantly different between the two groups.

### Logistic regression analysis

The variance inflation factors of the size ratio, neck ratio, and height/width ratio were >10 in the collinearity test. Thus, we excluded these variables from the multivariate logistic regression analysis. Univariate analysis showed that the crude ORs in the ISS group with the covariates of age >54 years, BMI >28 kg/m^2^, posterior-circulation aneurysm, and balloon angioplasty were 0.559 (95% CI 0.338 to 0.924; P=0.023), 0.399 (95% CI 0.161 to 0.994; P=0.048), 2.175 (95% CI 1.335 to 3.543; P=0.002), and 1.771 (95% CI 1.056 to 2.968; P=0.03), respectively, in comparison with the non-ISS group. After all covariates with P values <0.1 were entered into the multivariate analysis using the backward stepwise selection method, the adjusted ORs in the ISS group with covariates of age >54 years, BMI >28 kg/m^2^, posterior-circulation aneurysm, and balloon angioplasty were 0.464 (95% CI 0.274 to 0.785; P=0.006), 0.427 (95% CI 0.184 to 0.991; P=0.026), 2.895 (95% CI 1.732 to 4.838; P<0.001), and 1.992 (95% CI 1.162 to 3.414; P=0.037), respectively, in comparison with the non-ISS group (table 3).

**Table 3:** Logistic regression analysis

Characteristics	Univariate analysis			Multivariate analysis		
	P value	Crude HR	95% CI	P value	Adjusted HR	95% CI
Male	0.128	1.456	0.897 to 2.363	–	–	–
Age >54 years	**0.023**	0.559	0.338 to 0.924	**0.006**	0.464	0.274 to 0.785
BMI ≥28 kg/m^2^	**0.048**	0.399	0.161 to 0.994	**0.026**	0.427	0.184 to 0.991
Diabetes mellitus	0.721	1.165	0.504 to 2.697	–	–	–
Dyslipidemia	0.408	0.812	0.495 to 1.331	–	–	–
Hypertension	0.728	1.089	0.673 to 1.762	–	–	–
Pre-SAH	0.886	0.919	0.288 to 2.929	–	–	–
Pre-stroke	0.786	1.152	0.416 to 3.189	–	–	–
Coronary artery disease	0.677	1.195	0.517 to 2.765	–	–	–
Current smoking	0.225	1.456	0.794 to 2.671	–	–	–
Regular alcohol drinkers	0.777	1.098	0.575 to 2.096	–	–	–
Allergy	0.131	0.523	0.226 to 1.212	–	–	–
Number of PEDs used	0.864	0.937	0.446 to 1.969	–	–	–
PED associated with coils	0.813	1.060	0.653 to 1.721	–	–	–
Aneurysm location in posterior circulation	**0.002**	2.175	1.335 to 3.543	**P<0.001**	2.895	1.732 to 4.838
Aneurysm with lobulation	0.590	1.225	0.585 to 2.564	–	–	–
Aneurysm with daughter sac	0.943	0.967	0.389 to 2.408	–	–	–
Aneurysm in bifurcation	0.157	1.933	0.776 to 4.816	–	–	–
Aneurysm neck	0.109	1.020	0.995 to 1.046	–	–	–
Maximum diameter	0.124	1.018	0.995 to 1.042	–	–	–
Aneurysm height	0.164	1.023	0.991 to 1.057	–	–	–
Aneurysm width	0.058	1.022	0.999 to 1.046	–	–	–
Aneurysm perpendicular height	0.106	1.028	0.994 to 1.063	–	–	–
Aspect ratio	0.219	0.800	0.561 to 1.142	–	–	–
Height/width ratio	**0.040**	0.387	0.157 to 0.956	–	–	–
Bottle neck factor	0.578	0.810	0.385 to 1.702	–	–	–
Size ratio	**0.030**	1.081	1.008 to 1.160	–	–	–
Neck ratio	**0.006**	1.130	1.035 to 1.235	–	–	–
Parental artery diameter	0.557	1.089	0.819 to 1.447	–	–	–
Proximal artery diameter	0.485	1.099	0.843 to 1.434	–	–	–
Distal artery diameter	0.395	1.132	0.851 to 1.505	–	–	–
Difference between proximal and distal artery	0.929	0.986	0.732 to 1.330	–	–	–
Proximal/distal ratio	0.958	0.977	0.407 to 2.343	–	–	–
Mean artery diameter	0.382	1.147	0.843 to 1.561	–	–	–
Recurrent aneurysm	0.713	1.303	0.319 to 5.331	–	–	–
Symptomatic aneurysm	0.736	1.086	0.672 to 1.756	–	–	–
Multiple aneurysm	0.327	0.760	0.439 to 1.316	–	–	–
Unsatisfactory device deployment	0.160	1.923	0.772 to 4.79	–	–	–
Balloon angioplasty	**0.030**	1.771	1.056 to 2.968	**0.037**	1.992	1.162 to 3.414
Preinterventional operation	0.370	0.588	0.185 to 1.874	–	–	–
Last follow-up complete occlusion	0.494	0.782	0.386 to 1.584	–	–	–
Fusiform aneurysm	**0.004**	2.062	1.258 to 3.380	–	–	–
Drug withdrawal	0.116	1.641	0.932 to 2.903	–	–	–
Clopidogrel switched to ticagrelor	0.256	1.457	0.762 to 2.789	–	–	–

BMI, body mass index; ISS, in-stent stenosis; PED, Pipeline embolization device; SAH, subarachnoid hemorrhage.

### Dynamic changes in ISS

**Figure 1:**
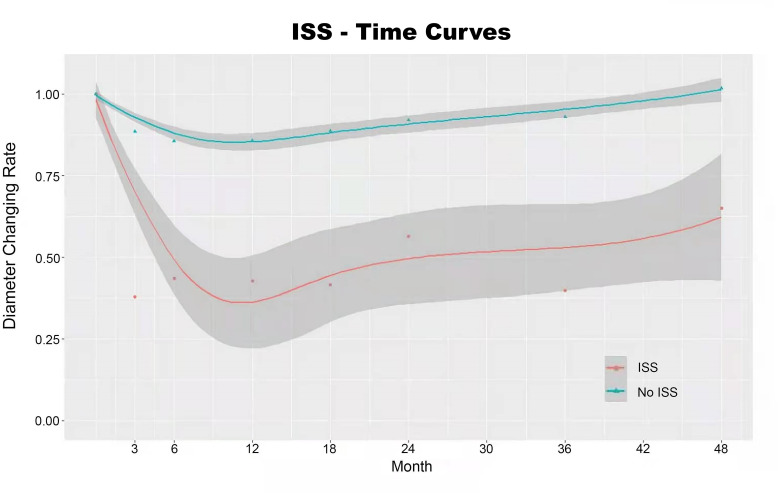
In-stent stenosis (ISS)–time curves in the ISS (n=69) and non-ISS (n=390) groups. The diameter changing rate was defined as 1 – stenosis rate. Both the ISS and non-ISS groups showed a trend towards a peak in endothelialization initiated by implantation of the Pipeline embolization device at 6–12 months, which rarely progressed after 12 months. ISS was reversed within 24 months after device implantation.

We constructed the subgroup ST-T curve (ISS group (n=69) versus non-ISS group (n=390); figure 1) to demonstrate the dynamic changes in parental artery diameter after PED implantation. We used the diameter changing rate (calculated as 1 − SR) for comparison of the original diameter (measured immediately after the procedure) of the parental artery and the follow-up diameter of the parental artery to standardize the variation in vessel diameter of each patient. The diameter changing rate calculated immediately after the procedure was 1 in both the ISS and non-ISS groups. The ST-T curve showed that simultaneous and progressive endothelialization initiated by PED implantation reached its peak at 6–12 months and rarely progressed after 12 months, while intimal hyperplasia progression of ISS was reversed within 24 months.

### Subgroup analysis

We conducted a subgroup analysis to compare the mean duration of follow-up between the resolution group and the non-resolution group. We conducted another subgroup analysis to identify differences in the resolution and progression to artery occlusion between patients with ISS who were receiving DAPT and those who were not, as well as a subgroup analysis to identify differences between responders and non-responders (according to the platelet function test) with respect to ISS. Finally, we evaluated the patients with ISS for evidence of proximal or distal ‘fish-mouthing’ and late distortion of the actual PED at follow-up angiography. The results of the subgroup analyses are presented in the online supplemental material.

## Discussion

In the present study, the incidence of ISS was approximately 15% after PED implantation for intracranial aneurysm. In the majority of patients in the ISS group, ISS developed into mild stenosis (53.2%), while ISS developed into parental artery occlusion in approximately 30% of patients. Previous studies have shown different incidence rates (1.1%–29.3%) of ISS after PED implantation.^12 14 16 22^ However, because of the very small number of total cases and lack of long-term dynamic follow-up data for ISS findings in those studies, it is difficult to conduct further analyses on factors affecting ISS. From our large sample of patients who underwent PED implantation and who had ISS, we found that age >54 years and a BMI >28 kg/m^2^ were protective factors for ISS, while balloon angioplasty and posterior-circulation aneurysm were risk factors for ISS.

There are marked differences in the current diagnostic criteria for ISS. ISS is commonly classified as mild (SR: 0–25%), moderate (SR: 25–50%), or severe (SR >50%). Some studies have defined ISS as a reduction in blood vessel diameter of ≥25%. In the present study, we defined the occurrence of ISS as an SR of ≥50%. This definition of ISS may be too conservative and potentially created a bias in favor of PED. However, we chose this definition because mild intimal hyperplasia after stenting is expected, desirable, and required to achieve aneurysm occlusion.^14^ Therefore, only more robust reactive stenosis, which is probably detrimental because of hemodynamically significant flow limitations, was graded as ISS.

Our finding that ISS was less common in older people is similar to previous studies. Sweid *et al* 23 reported that advanced age was associated with a decreased capacity for endothelial regeneration, which explains the lower ISS rate. Specifically, lack of endothelialization was found in the aging brain, which reduced the potential for neural stem cell proliferation and differentiation. However, aging is a double-edged sword for patients who undergo PED implantation because a lack of endothelialization is responsible for failed aneurysm occlusion, necessitating an extended follow-up time and aneurysm re-treatment.^24^

Patients who undergo balloon angioplasty are more likely to develop ISS after surgery. This may occur for two reasons. First, the basic cerebrovascular condition of these patients is poor, and local stenosis or atherosclerosis may be present. Moreover, blood vessels in the lesion area are relatively tortuous. Thus, the microcatheter cannot reach the finest landing area during surgery. In this situation, if balloon angioplasty is not used, it will be difficult to ensure that the stent adheres to the vessel wall to treat the aneurysm. Second, balloon angioplasty can cause mechanical damage to the blood vessel wall, leading to intensified local inflammation and subsequent excessive intimal hyperplasia.^23^ On the basis of our findings, we recommend using the PED to treat intracranial aneurysms. Nevertheless, neuroradiologists should evaluate the benefits and risks based on the condition of patients who have undergone balloon angioplasty. If balloon angioplasty is inevitable, long-term follow-up is necessary, and the antiplatelet drug treatment plan needs to be dynamically adjusted according to the results of angiographic follow-up.

In contrast to our findings, Chalouhi *et al* reported that anterior-circulation aneurysms, especially internal carotid artery aneurysms, were a risk factor for ISS after PED implantation (OR=3.1; P=0.03).^13^ In that study, the number of patients with posterior-circulation aneurysms was small. This might be because neuroradiologists were cautious about applying the PED to posterior-circulation aneurysms at that time, as the treatment of these aneurysms was not an original indication for PED implantation. However, indications for use of the PED have expanded with increasing data on its efficacy and safety. More recently, many studies have reported the feasibility of the PED for posterior-circulation aneurysm treatment.^25 26^ Although the proportion of aneurysms in the anterior circulation exceeded 80% in our study, considering that neuroradiologists remain cautious when deciding whether to use the PED to treat posterior-circulation aneurysms, we consider that this ratio was acceptable.

In the present study, the incidence of ISS after PED treatment for posterior-circulation aneurysms was higher than that for anterior-circulation aneurysms, and two patients died from complications caused by basilar artery infarction due to basilar artery occlusion after ISS. Additionally, posterior-circulation aneurysms are mostly dissecting aneurysms, and typically, dissecting aneurysms are often associated with a higher rate of parent artery stenosis.^27^ Moreover, some of the posterior-circulation aneurysms treated with the PED in the present study were large or giant vertebrobasilar fusiform aneurysms. These aneurysms often involve a long section of the parent artery, and there is often partial thrombosis in the aneurysm sac. To adequately cover the aneurysm, treatment requires the use of multiple PEDs with overlapping placement, as well as placement across vessels of different diameters.^28^ These factors all increase the risk of long-term postoperative ISS.

Interestingly, we found that a higher BMI was associated with decreased occurrence of ISS. To our knowledge, BMI has not been previously reported to affect ISS. Notably, West *et al* 29 found that restenosis after intracoronary stent placement was associated with reduced BMI, and the authors suggested that patients with a lower BMI have smaller coronary arteries than obese patients. Thus, we speculate that ISS after PED implantation may follow a similar course. Nevertheless, it remains unclear whether the higher BMI in the ISS group reflected a larger cerebral vessel diameter. Further studies are required to elucidate the reason for this finding and to explore the relationship between BMI, vessel diameter, and ISS risk.

For patients with ISS without symptoms, we prescribed atorvastatin (20 mg/day) if they had not completed 6 months of standard DAPT (aspirin combined with clopidogrel or ticagrelor). If they had completed 6 months of DAPT, we prescribed aspirin combined with atorvastatin until the next follow-up. For patients with ISS with symptoms, we prescribed atorvastatin (20 mg/day) and extended the duration of DAPT if they had not completed 6 months of DAPT. We also recommended that the patients resumed DAPT and added atorvastatin until the next follow-up if they had completed 6 months of DAPT. According to our subgroup analysis, patients with ISS had a higher resolution rate if they extended their dose or resumed DAPT (n=4 (7.8%) vs n=5 (27.8%), respectively; P=0.045). These data suggest that restoration or prolongation of DAPT contributes to ISS resolution, although the exact mechanism remains unclear. For patients with ISS who developed parental artery occlusion, there was no difference between the aspirin group and the DAPT group (n=12 (22.6%) vs n=6 (26.1%), respectively; P=0.240).

Our results show that tissue growth in normal appearing PED occurs in the majority of patients with ISS. However, further analysis revealed that all patients who developed parental artery occlusion had late distortion of the PED. Furthermore, in two patients who died, the type of ISS involved distal distortion of the PED, suggesting that ISS caused by late flow diverter distortion is more likely to have catastrophic consequences and requires particular attention from clinicians, which is consistent with previous studies.^12 14^

Our study demonstrated that ISS is usually asymptomatic, with only a small number of patients (7/69 (10.1%)) experiencing symptoms (eg, dizziness, hemianopsia, hemidysesthesia, and hemiplegia). ISS was reversible in most patients (51/69 (73.9%)) and sometimes improved or completely recovered without retreatment (9/69 (13.0%)), while only a small number of patients worsened (7/69 (10.1%)). Only one patient (1/69 (1.4%)), who was originally diagnosed with asymptomatic ISS, developed symptoms. This patient stopped using antiplatelet drugs without authorization because of a femur fracture 10 months after PED treatment for basilar aneurysm, and had a brainstem infarction during the 12-month follow-up. Other patients with asymptomatic in-stentstenosis (ISS) who took their prescribed medication did not develop new symptoms during follow-up. Thus, we suggest that patients with asymptomatic ISR identified during follow-up have a low risk of progression to symptomatic ISR when treated with appropriate antiplatelet drugs and statins.

Because of the relatively slow progress of ISS, not all cases were detected during the first angiographic follow-up, suggesting that ISS does not necessarily occur within 3–6 months after PED implantation. On the basis of our ST-T curve, the intimal hyperplasia initiated by PED implantation peaked and developed aggressively at 6–12 months after implantation. Subgroup analysis showed a significant difference in average follow-up duration between the resolution group and the non-resolution group. According to the ST-T curve, patients who developed ISS showed a clear trend towards resolution at 24 months after PED implantation, which fits with our statistical analyses. For these reasons, we strongly recommend at least 2 years of postoperative angiographic follow-up to identify ISS and to dynamically evaluate the degree of intimal hyperplasia in patients undergoing PED placement. Even if ISS leads to complete blood vessel occlusion, the cerebral circulation can have abundant collateral circulation, and ISS will not cause serious symptoms as it occurs very slowly. Note that current treatment for ISS remains empirical.^30^ For patients with ISS, close follow-up and long-term antiplatelet therapy are recommended. If the blood vessel is nearly completely occluded and causes symptoms, angioplasty, stent reimplantation, or even bypass surgery can be used to relieve blood vessel narrowing.

### Limitations

Our study has several limitations. First, this was a single-center study, and the results cannot be generalized to other patient groups. However, a single-center design was chosen because we needed to control the main confounding factors. Second, all of the angiographic parameters were obtained by manual measurement, Thus, measurement errors might have biased the final results. Third, we used self-reported data to assess medication compliance during follow-up, which might have caused bias. Finally, there may be unknown confounding factors that we did not consider in our analysis that could have influenced our results.

## Conclusion

We found that ISS after PED implantation is a common and self-limiting complication. Patients with posterior-circulation aneurysms and balloon angioplasty were more likely to develop ISS. Patients aged >54 years or with a BMI >28 kg/m^2^ had a lower risk of ISS occurrence and progression. Intimal hyperplasia initiated by PED implantation reached its peak at 6–12 months, and ISS progression was reversed within 24 months.

10.1136/jnis-2022-019680.supp2 Supplementary data

## Data Availability

Data are available upon reasonable request. The authors agree to share data upon reasonable request. Any data from this study are available by contacting the corresponding author.
