# Supplementary material for: Long-term outcomes and dynamic changes of in-stent stenosis after Pipeline embolization device treatment of intracranial aneurysms
Source: J Neurointerv Surg. 2023 Jan 23;15(12):1187–93. doi: 10.1136/jnis-2022-019680 (PMC10715560; doi:10.1136/jnis-2022-019680)

BMJ Publishing Group Limited (BMJ) disclaims all liability and responsibility arising from any reliance placed on this supplemental material which has been supplied by the author(s)

Definition of anterior-circulation aneurysm and posterior-circulation aneurysm

Aneurysms located at the internal carotid artery, anterior communicating artery, posterior communicating artery, anterior cerebral artery, and middle cerebral artery were defined as anterior-circulation aneurysms. Aneurysms located at the vertebral artery, basilar artery, posterior inferior cerebellar artery, and posterior cerebral artery were defined as posterior-circulation aneurysms.

Definition of aneurysm and parental artery morphology parameters

The morphology parameters assessed in this study were defined as follows:

Aspect ratio: aneurysm height ÷ aneurysm width;

size ratio: maximum diameter of aneurysm ÷ parental artery diameter;

height/width ratio: perpendicular height of aneurysm ÷ aneurysm width;

bottle/neck factor: aneurysm neck length ÷ aneurysm width;

neck ratio: aneurysm neck length ÷ parental artery diameter;

mean artery diameter: (distal artery diameter + proximal artery diameter) ÷ 2;

artery difference: proximal artery diameter − distal artery diameter;

proximal-distal ratio: proximal artery diameter ÷ distal artery diameter.

All parameters were measured and calculated based on original digital subtraction angiography photographs.

Study Size Calculation

No sample size calculation was performed and the sample size was established by the time window of the study.

Platelet function test statement

The experimental diagnostic center at our hospital does not support PRU (P2Y12 reaction units) testing at present. Instead, all patients in our study underwent platelet function monitoring 1 day before PED placement. Platelet function was assessed by standard light transmittance aggregometry (LTA) to measure platelet aggregation. Light transmittance aggregometry was conducted using platelet-rich plasma using the turbidimetric method in a 4-channel aggregometer (AG800; Techlink Biomedical, Inc., Beijing, China). Maximal platelet aggregation (MPA) was defined as the percentage change in light transmittance. Subsequently, non-responders were defined as having an MPA response to ADP (adenosine diphosphate) of >50%. For those patients, clopidogrel was switched to one dose of ticagrelor (180 mg) before the procedure, followed by twice daily doses of ticagrelor (45 mg) after the procedure combined with aspirin (100 mg) for 6 months.

Statement for generation of PED used in this study

Pipeline Embolization Device and Pipeline Flex Embolization Device were used in our study without include PED Shield (Pipeline embolization device with Shield technology).

PED implantation procedure

The PED was delivered and deployed through a Marksman™ microcatheter (Medtronic, Irvine, CA) or an Excelsior™ XT-27™ microcatheter (Stryker, Kalamazoo, MI). PED-assisted coiling was considered if there was (a) a risk of shortening and displacement of the PED after release or (b) rapid blood flow (jet) at the aneurysmal neck on angiography, which was expected to pose a high risk of recurrence and postoperative bleeding with FD implantation alone. The brands of coil included Axium™ (Medtronic, Dublin, Ireland), Microplex™ (Microvention, Aliso Viejo, CA), Target™ (Stryker, Kalamazoo), and Orbit™ (Johnson & Johnson, New Brunswick, NJ). When full vessel wall apposition was not

achieved, stent massage or balloon angioplasty was performed.

Antiplatelet therapy after PED implantation

The duration of dual antiplatelet therapy (DAPT) after PED implantation was 6 months for aspirin (100 mg/day) combined with clopidogrel (75 mg/day), and aspirin (100 mg/day) was continued for at least 1 year. For patients with inadequate platelet inhibition with clopidogrel (platelet function test showed maximal platelet aggregation of >50%), clopidogrel was switched to one dose of ticagrelor (180 mg) before the procedure, followed by twice daily doses of ticagrelor (45 mg) for 6 months after the procedure. In fact, some patients with poor adherence spontaneously withdrew the antiplatelet drugs. We have added these data to our revised manuscript. The duration and type of antiplatelet therapy have been included in the manuscript and analyzed as variables

Analysis for drug withdraw between Non-ISS and ISS group

	No-ISS	ISS	Total	P value
Drug withdraw	82 (21.03%)	21 (30.43%)	103 (22.44%)	0.116

Univariate logistic regression	OR	95% CI	P value
Drug withdraw	1.641	0.932—2.903	0.116

Analysis for clopidogrel switched to ticagrelor between Non-ISS and ISS group

	No-ISS	ISS	Total	P value
Clopidogrel switched to ticagrelor	58 (14.9%)	14 (20.3%)	72 (15.7%)	0.254

Univariate logistic regression	OR	95% CI	P value
Clopidogrel switched to ticagrelor	1.457	0.762—2.789	0.256

Subgroup analysis

Subgroup analysis for the average duration of follow-up between resolution group vs non-resolution group in ISS patients

There was a significant difference between the resolution group and the non-resolution group (25 [14–36] months vs. 14 [9–20] months; P = 0.005) in terms of the average duration of follow-up in patients with ISS. According to the ST-T curve, patients who developed ISS showed a clear trend toward resolution 24 months after PED implantation, which is in line with the statistical data.

Subgroup analysis for the difference in resolution and progression to artery occlusion between ISS patients who were on DAPT and non-DAPT

Patients who had ISR had a higher rate of resolution if they extended their dose or resumed DAPT (4 [7.8%] vs. 5 [27.8%]; P = 0.045). For patients with ISS who developed parental artery occlusion, there was no significant difference between the aspirin group and the DAPT group (12 [22.6%] vs. 6 [26.1%]; P = 0.240).

	Non-DAPT	DAPT	Total	P value
ISS to resolution	4 (7.8%)	5 (27.8%)	9 (13.0%)	**0.045**
ISS to occlusion	12 (22.6%)	6 (37.5%)	18 (26.1%)	0.240

Subgroup analysis for the difference between responders and non-responders (according to the platelet function test) respect to ISR

There was no difference between responders and non-responders with respect to ISR in the subgroup analysis (55 [14.2%] vs. 14 [19.4%]; P = 0.254)

	Responders	Non-responders	Total	P-value
In stent-stenosis	55 (14.2%)	14 (19.4%)	69 (15.0%)	0.254

Evaluation of the ISS patients for proximal or distal "fishmouthing" and diffuse or focal mechanical distortion of the actual Pipeline Embolization Device

Types of in-stent stenosis	Numbers of patients (%)	Numbers of patients developed to parental artery occlusion (%)
Tissue growth in normal appearing PED	40 (57.97%)	0 (0.00%)
Proximal distortion (fish-mouthing) of the PED	9 (13.04%)	8 (44.4%)
Distal distortion (fish-mouthing) of the PED[#]	14 (20.29%)	8 (44.4%)
Distortion of the mid-portion of the PED	3 (4.35%)	1 (5.56%)
Tissue growth with distal distortion of the PED	2 (2.90%)	1 (5.56%)
Tissue growth with proximal distortion of the PED	1 (1.45%)	0 (0.00%)
Total	69 (100%)	18 (100%)

[#]The type of ISS was evaluated as distal distortion (fish-mouthing) of the PED in two patients that died.

After further back-to-back blinded review of follow up angiography of 69 patients with ISS, we found that 40 (57.97%) patients presented with tissue growth in normal appearing PED, nine (13.04%) patients had proximal distortion (fish-mouthing) of the PED, 14 (20.29%) patients had distal distortion (fish-mouthing) of the PED, and three (4.35%) patients had distortion of the mid-portion of the PED. Furthermore, two (2.90%) patients had normal tissue growth with distal distortion of the ISS, and one (1.45%) patient had tissue growth with proximal distortion of the PED. For the 18 patients who developed parental artery occlusion, dynamic assessment of postoperative follow-up angiography showed eight (44.44%) patients with proximal distortion (fish-mouthing) of the PED, eight (44.44%) patients with distal distortion (fish-mouthing) of the PED, and one (5.56%) patient with distortion of the mid-portion of the PED. One (5.56%) patient presented with normal tissue growth with distal distortion of the PED.

Interestingly, we found that nearly 2/3 of patients presenting with late stent distortion had their procedure between 2015–2018, with many Chinese physicians having only 1–3 years of

experience in using PEDs. At that time, some of the physicians used the biaxial system (e.g., a 6F guiding catheter combined with a Marksman micro catheter) to deliver and deploy the PEDs. Although the biaxial system is the classic approach in cerebrovascular interventions, it has poor support in curved vessels, which can lead to poor PED apposition. Furthermore, even if the PED achieves an adequate apposition, the operator has to perform more pushing and pulling maneuvers, which can increase the risk of irreversible damage to the PED structure (e.g., twisting in the middle of the stent). Finally, the distal or proximal part of the PED can be occasionally or inevitably placed into the curved vessel area of patients with a tortuous blood vessel. All these factors can lead to late distortion of the PED.

When selecting the PED size, the operator will often measure the proximal and distal parental artery diameters. However, to achieve adequate proximal wall apposition, the operator often prefers to accommodate the proximal parental artery diameter. This inevitably results in a ‘mismatch’ between the PED size and the distal vessel diameter, while the pressure from stent expansion can result in intima damage at the distal part of the stent. We believe that these factors may account for the greater number of patients with distal distortions than proximal distortions.

To prevent the late mechanical distortion of the PED, we recommend that the operator use a triaxial system to deliver and release the PED (e.g., the Neuron MAX 088 catheter combined with the Navien intracranial support catheter and the Phenom-27 microcatheter). If poor wall apposition is identified intraoperatively, the microguide wire massage stent should be used carefully (careless handling can cause damage to the proximal part of the stent and may lead to proximal distortion), and balloon-angioplasty or even further stent deployment (e.g., Neuroform EZ) should be used to achieve adequate apposition. Note that this additional manipulation is also associated with increased risk of ISS.

Supplementary Table 1-1. Aneurysm characteristics of patients after PED treatment

**Characteristics**	Non-ISS (n=390)	ISS (n=69)	Total (n=459)	P value
**Aneurysm location**				**0.008**
**ACA**	2 (0.5%)	0 (0%)	2 (0.4%)	
**AComA**	1 (0.3%)	0 (0%)	1 (0.2%)	
**BA**	10 (2.6%)	2 (2.9%)	12 (2.6%)	
**ICA**	298 (76.4%)	40 (58%)	338 (73.6%)	
**MCA**	8 (2.1%)	2 (2.9%)	10 (2.2%)	
**PCA**	2 (0.5%)	1 (1.4%)	3 (0.7%)	
**PComA**	2 (0.5%)	0 (0%)	2 (0.4%)	
**PICA**	0 (0%)	2 (2.9%)	2 (0.4%)	
**VA**	67 (17.2%)	22 (31.9%)	89 (19.4%)	
**Aneurysm position**				0.390
**middle**	11 (2.9%)	2 (2.9%)	13 (2.8%)	
**left**	207 (53.1%)	30 (43.5%)	237 (51.6%)	
**right**	172 (44.1%)	37 (53.6%)	209 (45.5%)	
**Aneurysm type**				**0.001**
**saccular**	316 (81.03%)	44 (63.77%)	360 (78.43%)	
**fusiform**	74 (18.97%)	25 (36.23%)	99 (21.57%)	
**Aneurysm in bifurcation**	16 (4.1%)	5 (7.2%)	21 (4.6%)	0.401
**Aneurysm with lobulation**	43 (11.0%)	8 (11.6%)	51 (11.1%)	0.890
**Aneurysm with daughter sac**	28 (7.2%)	5 (7.2%)	33 (7.2%)	1.000
**Multiple aneurysms**	116 (29.7%)	18 (26.1%)	134 (29.2%)	0.538
**Symptomatic aneurysms**	172 (44.1%)	35 (50.7%)	207 (45.1%)	0.308
**Recurrent Aneurysms**	7 (1.8%)	2 (2.9%)	9 (2.0%)	0.890

ACA: anterior cerebral artery; AComA: anterior communicating artery; MCA: middle cerebral artery; ICA: internal carotid artery; VA: vertebral artery; BA: basilar artery; PICA: posterior inferior cerebellar

artery; PComA: posterior communicating artery; PCA: posterior cerebral artery

Supplementary Table 1-2. Aneurysm characteristics of patients after PED treatment

**Characteristics**	Non-ISS (n=390)	ISS (n=69)	Total (n=459)	P value
**Unsatisfiable Device Deployment**	11 (2.8%)	5 (7.2%)	16 (3.5%)	0.136
**Balloon angioplasty**	68 (17.4%)	21 (30.4%)	89 (19.4%)	**0.012**
**PED associated with coiling**	146 (37.4%)	29 (42.0%)	175 (38.1%)	0.469
**Used PED>1**	46 (11.8%)	9 (13.0%)	55 (12.0%)	0.769
**Aneurysm Neck**	6.69 (4.45—11.10)	10.10 (6.52—15.75)	7.04 (4.50—11.60)	**P<0.001**
**Maximum Diameter**	10.40 (6.29—16.70)	13.30 (9.07—22.20)	10.90 (6.40—17.10)	**0.003**
**Aneurysm Height**	7.37 (4.76—11.80)	7.87 (6.57—14.45)	7.43 (4.95—12.10)	**0.025**
**Aneurysm Width**	8.48 (4.81—14.63)	12.9 (6.80—20.95)	9.02 (5.07—15.30)	**P<0.001**
**Aneurysm Perpendicular Height**	6.96 (4.50—11.23)	7.87 (5.83—14.25)	7.18 (4.68—11.60)	**0.017**
**Parental Artery Diameter**	3.64 (3.16—4.17)	3.82 (3.30—4.36)	3.66 (3.17—4.19)	0.304
**Proximal Artery Diameter**	3.91 (3.45—4.49)	4.00 (3.15—4.79)	3.92 (3.42—4.53)	0.890
**Distal Artery Diameter**	3.43	3.49	3.44	0.327

	(3.04—3.86)	(2.82—4.22)	(2.98—3.88)	
**Mean Artery Diameter**	3.68 (3.21—4.08)	3.80 (3.19—4.36)	3.68 (3.21—4.09)	0.702
**Difference between Proximal and Distal Artery**	0.45 (0.09—1.01)	0.49 (0.06—1.08)	0.46 (0.09—1.01)	0.882
**Proximal/Distal Ratio**	1.13 (1.03—1.30)	1.12 (1.02—1.34)	1.13 (1.03—1.31)	0.900
**Aspect Ratio**	1.07 (0.74—1.59)	0.94 (0.66—1.50)	1.04 (0.73—1.59)	0.141
**Height/Width Ratio**	0.89 (0.74—1.02)	0.81 (0.64—0.95)	0.87 (0.72—1.01)	**0.012**
**Bottle Neck Factor**	0.94 (0.69—1.00)	1.00 (0.68—1.00)	0.95 (0.69—1.00)	0.865
**Size Ratio**	2.87 (1.64—4.73)	3.56 (2.18—6.32)	3.00 (1.73—4.81)	**0.007**
**Neck Ratio**	1.91 (1.15—3.02)	2.93 (1.56—4.40)	1.99 (1.19—3.40)	**P<0.001**

PED: Pipeline Embolization Device

Supplementary Figure 1 Flow Chart

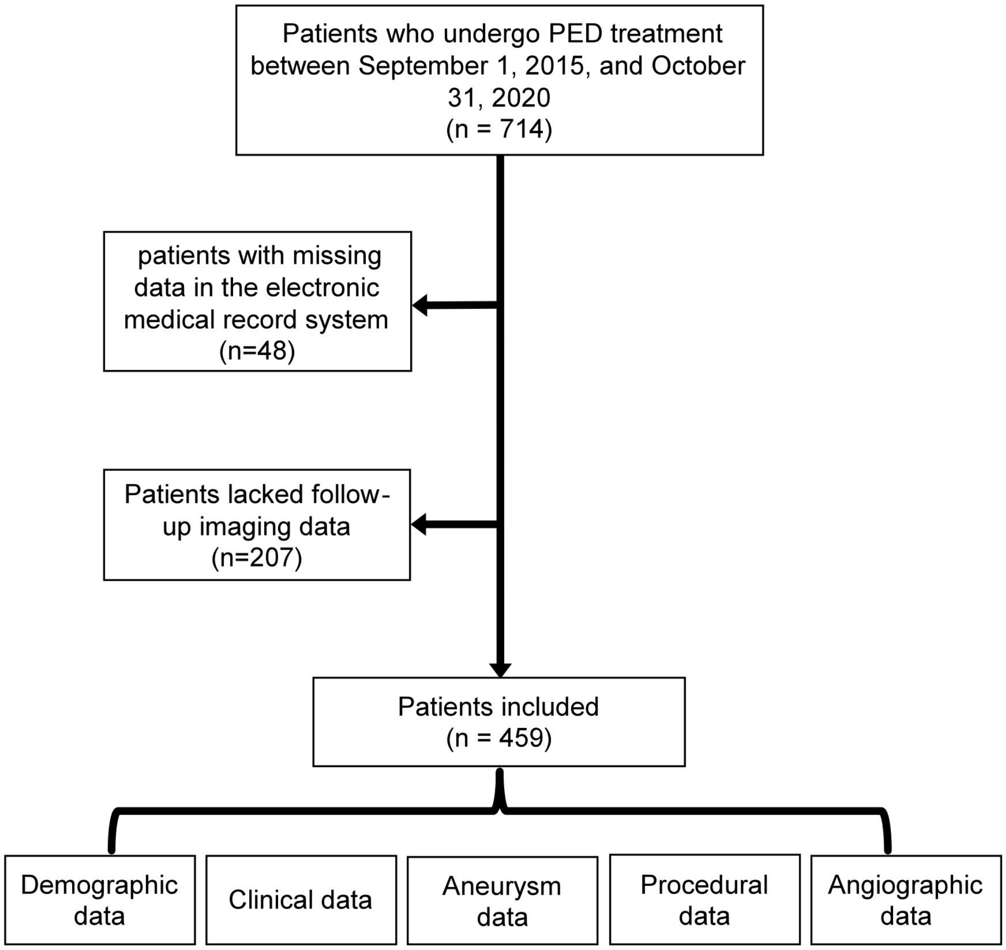

Supplement: Supplementary data [file jnis-2022-019680supp001.pdf]
